# Whole-Exome Sequencing Identifies Recurrent Germline-Associated and Somatic Variants in Oral Squamous Cell Carcinoma from Southwest India

**DOI:** 10.3390/biomedicines14061346

**Published:** 2026-06-15

**Authors:** Hafeeda Kunhabdulla, Riaz Abdulla, Rohan Thomas, Dhanya Shetty, Mohammed S. Mustak, Ranajit Das

**Affiliations:** 1 Department of Oral and Maxillofacial Pathology and Microbiology, Yenepoya Dental College, Yenepoya (Deemed to Be University), Deralakatte, Mangalore 575018, Karnataka, India; hafeedadent@gmail.com; 2 Department of Surgical Oncology, Zulekha Yenepoya Institute of Oncology, Yenepoya (Deemed to Be University), Mangalore 575018, Karnataka, India; rohanthomas@gmail.com; 3 Center for Systems Biology and Molecular Medicine (CSBMM), Yenepoya Research Centre, Yenepoya (Deemed to Be University), Mangalore 575018, Karnataka, India; dhanyashetty090909@gmail.com; 4 Department of Applied Zoology, Mangalore University, Mangalagangothri, Mangalore 575199, Karnataka, India; msmustak@gmail.com

**Keywords:** oral squamous cell carcinoma, south west coast of India, mutation

## Abstract

**Background:** Oral squamous cell carcinoma (OSCC) remains a major public health challenge, particularly in South Asian populations where environmental exposures such as tobacco and areca nut consumption contribute significantly to disease burden. Although genomic studies have improved understanding of oral cancer biology, population-specific genomic data from high-risk Indian populations remain limited. This study aimed to characterize the genomic landscape of OSCC using whole-exome sequencing (WES) of fresh biopsy specimens obtained from patients residing along the southwest coast of Karnataka, India. **Methods:** Paired tumor and adjacent normal tissues from ten OSCC samples (total *n* = 20 samples) were subjected to WES to identify somatic and germline-associated variants. Matched tumor–normal comparative analysis, variant annotation, and population frequency assessment using established genomic databases, including gnomAD, were performed to characterize the mutational profile. The findings were further compared with a previously analyzed regional cohort comprising 66 OSCC patients to evaluate recurrence patterns and population relevance. **Results:** The analysis identified a broad background of recurrent germline-associated variants alongside a comparatively limited number of tumor-specific somatic mutations, consistent with the expected predominance of constitutional genetic variation relative to acquired coding alterations in tumor samples. Recurrent variants were observed in genes associated with DNA repair, immune signaling, inflammatory responses, and pharmacogenomic pathways, including *XRCC1*, *ITPKB*, *ABCB1*, and *OPRM1*, whereas somatic alterations were primarily detected in established cancer-associated genes such as *TP53*, *CDKN2A*, and *TERT*. **Conclusions:** Several recurrent variants demonstrated high frequencies in South Asian populations, suggesting that they may represent recurrent population-associated variants of potential biological or pharmacogenomic relevance that require validation in larger cohorts. KEGG pathway enrichment analysis identified pathways related to cancer, chemical carcinogenesis, metabolic regulation, and xenobiotic response. Overall, these findings provide preliminary insights into the population-specific genomic characteristics of OSCC in this regional cohort and highlight the need for larger validation studies to determine the biological significance and reproducibility of these findings.

## 1. Introduction

Oral squamous cell carcinoma (OSCC) is the most common malignancy of the oral cavity and a leading cause of cancer-related morbidity and mortality worldwide [1,2]. Despite advances in surgery, radiotherapy, and systemic therapy, OSCC continues to demonstrate high rates of recurrence, treatment resistance and substantial inter-patient heterogeneity, underscoring the need for improved molecular stratification and precision oncology approaches [1,3,4,5].

The burden of OSCC is particularly pronounced in South and Southeast Asia, where region-specific environmental exposures such as smokeless tobacco use, areca nut consumption, and chronic mucosal irritation play a major etiological role [2,4,5]. In addition to these environmental factors, accumulating evidence suggests that host genetic susceptibility and inflammatory responses significantly influence disease initiation, progression, and therapeutic outcomes, highlighting the importance of population-specific genomic investigations [6,7].

Next-generation sequencing (NGS) technologies have enabled comprehensive characterization of the genomic landscape of OSCC, revealing alterations in pathways related to cell cycle regulation, DNA damage repair, immune modulation, and xenobiotic metabolism [3,8]. Large-scale initiatives such as The Cancer Genome Atlas (TCGA) have identified recurrent somatic driver mutations in head and neck squamous cell carcinoma; however, these datasets are predominantly derived from Western populations and may not fully represent the genomic architecture of OSCC in high-risk Indian cohorts [3,9]. This underrepresentation limits the generalizability of existing biomarkers and highlights the need for population-specific genomic investigations to better understand regionally relevant disease biology.

In our previous work, whole-exome sequencing of formalin-fixed paraffin-embedded (FFPE) OSCC tissues revealed a distinct mutational landscape enriched for immune-related, pharmacogenomic, and DNA repair–associated genes, with limited overlap with canonical TCGA-HNSCC drivers [10]. Recurrent alterations in genes such as *ABCB1*, *XRCC1*, *TLR1*, and *IL6* suggested the presence of a stable biological core underlying OSCC in this population [10,11]. Although FFPE-based sequencing provides valuable retrospective insights and facilitates correlation with clinical outcomes, formalin-induced DNA fragmentation and sequence artefacts may limit sensitivity for certain variant classes and reduce genomic resolution [12,13].

To address these limitations, the present study employs whole-exome sequencing to achieve improved resolution of genomic alterations. Comparative analysis demonstrates retention of previously identified core genes while also identifying additional variants enriched in pathways related to DNA repair, drug metabolism, and immune signaling, thereby refining the genomic landscape of OSCC and enhancing biological interpretation [14,15,16].

In this context, the present study aims to characterize the genomic landscape of OSCC using whole-exome sequencing and to evaluate the relative contribution of germline and somatic variants, with the goal of improving understanding of population-specific disease biology and improving understanding of population-specific genomic variation in OSCC.

## 2. Materials and Methods

### 2.1. Sample Collection

Initially, twenty-two biopsy specimens were collected from patients diagnosed with oral squamous cell carcinoma (OSCC) residing along the southwest coast of India. The samples were obtained between 2024 and 2026 following approval from the Institutional Ethics Committee (Protocol No: YEC-1/2023/131). Written informed consent was secured from all participants, and all procedures adhered to the Declaration of Helsinki and approved institutional guidelines.

Of the 22 samples (11 pairs) initially collected, only those that satisfied the DNA quantity and quality criteria for whole-exome sequencing were included in the subsequent analysis. H&E-stained sections from each specimen were reviewed microscopically by an experienced oral pathologist to assess tumor cell adequacy and integrity. Tumor-rich and adjacent normal regions were manually marked prior to DNA extraction. Ultimately, ten paired tumor and adjacent normal tissue samples (*n* = 20) with sufficient DNA integrity and concentration (>20 ng/µL) were selected for sequencing.

### 2.2. Whole Exome Sequencing

Whole exome sequencing (WES) was performed to characterize the mutational profile of OSCC samples. Paired tumor and adjacent normal tissues were collected from ten individuals (total *n* = 20 samples), with each pair representing matched case (tumor) and control (adjacent normal) tissues from the same individual. Clinical characteristics of the study participants are summarized in Table 1.

WES libraries were prepared using the Twist Exome 2.0 kit. Genomic DNA was extracted from all tissue samples, and only samples with a DNA concentration ≥20 ng/μL were included for downstream sequencing. Sequencing was carried out on the Illumina NovaSeq 6000 platform, generating 150 bp paired-end reads (PE150). Raw sequencing reads obtained from fresh biopsy-derived DNA underwent adapter trimming and quality filtering to produce high-quality reads. The filtered reads were aligned to the human reference genome (GRCh37) using the Burrows–Wheeler Aligner (BWA) with default parameters [17]. Subsequent processing of aligned BAM files followed established best-practice pipelines for refinement and variant calling. Variants were identified and stored in VCF format.

Functional annotation of detected variants was performed using Ensembl’s Variant Effect Predictor (VEP) [18] to determine their predicted biological consequences. A matched tumor–normal comparative analysis pipeline was employed to distinguish somatic mutations from germline variants, with particular emphasis on alterations potentially contributing to tumorigenesis. Variant calling and classification were performed using established sequencing quality parameters, including minimum read depth (≥20×), mapping quality (MQ ≥ 40), genotype quality (GQ ≥ 30), and variant allele frequency (VAF) thresholds. Whole-exome sequencing generated approximately 6.6–13.2 Gb of high-quality trimmed sequencing data per sample following quality filtering. Sequencing libraries passed internal quality control assessments, including insert-size estimation, read-quality filtering, and library quantification prior to downstream alignment and variant calling. Processed reads were aligned to the GRCh37 human reference genome using BWA following GATK best-practice recommendations.

Germline variants were defined as variants detected in both tumor and matched adjacent normal tissues with consistent allele representation (typically VAF ~40–60% for heterozygous variants and >90% for homozygous variants), whereas somatic variants were identified as tumor-specific alterations absent in matched normal samples. Variants with low read support, poor mapping quality, low genotype confidence, strand bias, or features suggestive of sequencing artefacts were excluded from downstream analysis. Common polymorphisms were further annotated using dbSNP and gnomAD population databases, including South Asian allele frequency datasets, to distinguish recurrent population-associated variants from rare potentially functional alterations. Sequencing quality assessment included evaluation of mean on-target exome coverage, percentage of target bases covered at ≥20× depth, mapping efficiency, duplicate read percentage, median insert size, and overall sequencing quality metrics to ensure consistency and reliability across all samples. Final processed outputs included FASTQ, BAM, VCF, and annotated variant files, as provided in the sequencing report from NCGM.

### 2.3. Identification of the Mutational Landscape

Comprehensive annotation of identified variants was conducted using multiple clinically relevant databases and computational prediction tools to classify variant types and evaluate their pathogenicity. Tumor samples were systematically compared with their matched adjacent normal counterparts to identify somatic mutations and distinguish recurrent germline-associated variants, particularly those previously reported in relation to drug response, DNA repair, immune signaling, and other biologically relevant pathways. The dbSNP database was utilized to catalog known genetic variants and obtain reference information, while ClinVar [19,20,21] was consulted to identify variants previously classified as pathogenic, likely pathogenic, or associated with pharmacogenomic traits. In addition, in silico prediction tools such as PolyPhen-2 [22] were employed to assess the potential functional impact of missense variants, and the OMIM^®^ database [23] was examined to determine reported associations between identified variants and known genetic disorders. Given that adjacent normal tissues were utilized as controls, the possibility of molecular alterations related to field cancerization cannot be excluded, and, therefore, variants detected in adjacent tissues should be interpreted cautiously. Therefore, variants detected in both tumor and adjacent normal tissues were cautiously interpreted as recurrent germline-associated or population-associated recurrent variants rather than definitive tumor-driving somatic alterations. All statistical analyses were performed in R v4.5.1 [24,25].

Notably, given that adjacent normal tissues were utilized as controls, the potential for field cancerization cannot be entirely dismissed. Consequently, variants identified in both tumor and adjacent normal tissues were cautiously interpreted as recurrent germline-associated or population-associated variants, rather than definitive cancer-driving alterations.

## 3. Results

### 3.1. Overall Variant Distribution and Overlap

**Figure 1:**
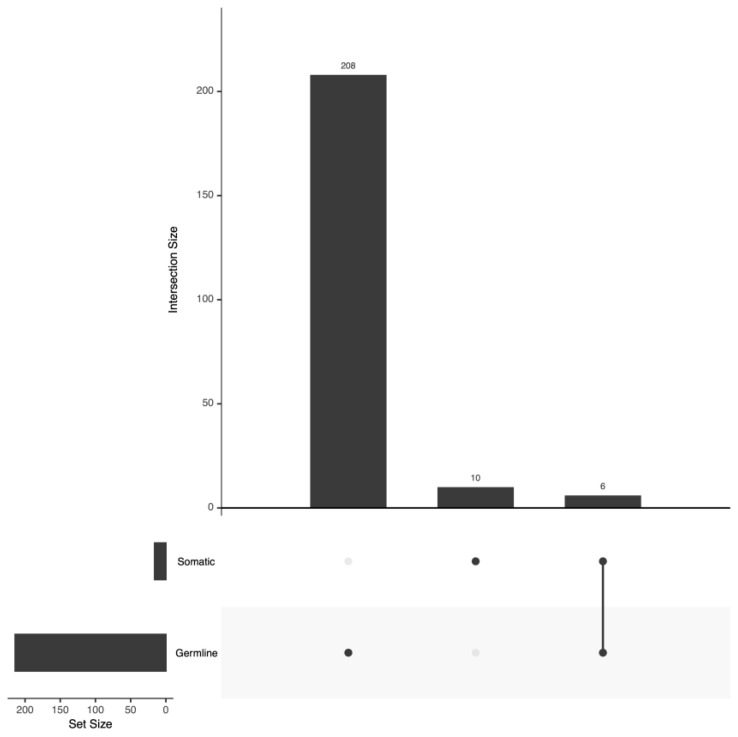
Overlap between germline and somatic variants identified in OSCC samples. UpSet plot showing the distribution and intersection of variants detected in paired tumor and adjacent normal tissues from 10 OSCC patients. A total of 208 germline and 16 somatic variants were identified, with 6 genes shared between both categories. The majority of variants were unique to the germline compartment, indicating a substantially higher background germline variant load compared to tumor-specific somatic alterations.

Whole exome sequencing of 10 paired OSCC tumor and adjacent normal tissues identified a total of 208 germline variants and 16 somatic variants, of which 6 genes were shared between germline and somatic datasets (Figure 1). The UpSet analysis demonstrated that the majority of variants were unique to the germline compartment, with comparatively fewer tumor-specific somatic alterations. This indicates a substantially higher background germline variant burden relative to acquired somatic mutations within the analyzed cohort.

### 3.2. Variant Burden per Patient

**Figure 2:**
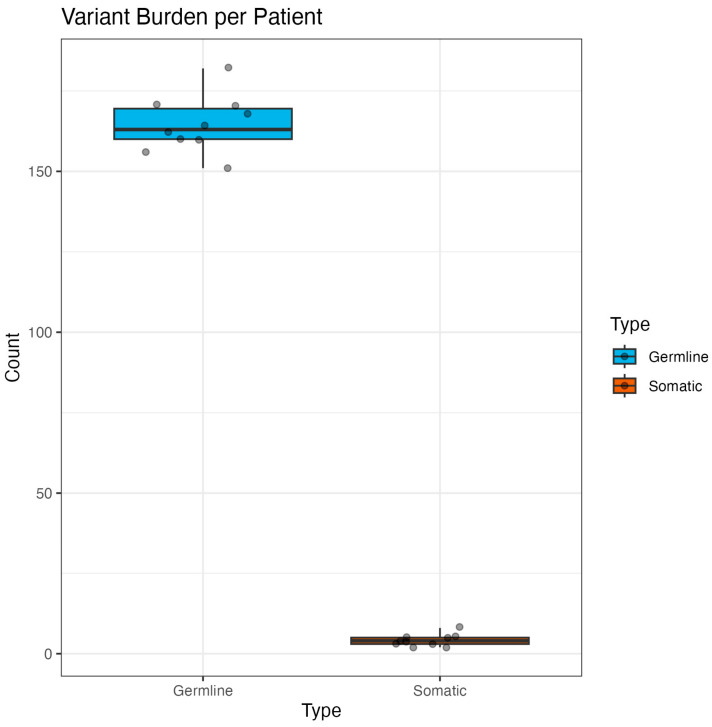
Variant burden per patient in germline and tumor samples. Boxplot illustrating the number of variants detected per patient in germline (adjacent normal tissue) and somatic (tumor tissue) compartments. Germline samples exhibited a markedly higher median variant count (~160–170 variants per individual) compared to somatic samples (~3–5 variants per tumor). Inter-individual variability was observed in germline variants, whereas somatic mutation counts remained consistently low across patients.

Per-patient variant burden analysis showed a markedly higher number of germline variants (median ~160–170 variants per individual) compared to somatic variants (median ~3–5 variants per tumor sample) (Figure 2). Germline variant counts displayed moderate inter-individual variability, whereas somatic mutation counts were consistently low across cases. This contrast highlights the relative sparsity of high-confidence somatic coding mutations in this cohort.

### 3.3. Frequently Mutated Germline Genes

**Figure 3:**
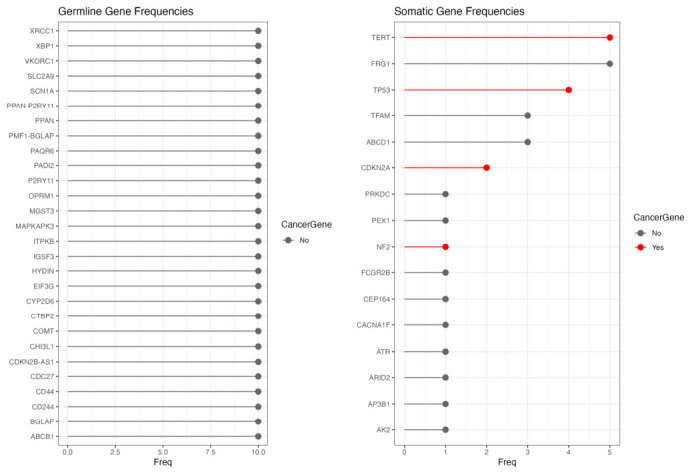
Frequency distribution of recurrently mutated genes in OSCC samples. Bar/lollipop plot showing the frequency of recurrently mutated genes across the cohort. Germline variants were recurrent in genes including *XRCC1*, *CDKN2B-AS1*, *ABCB1*, *OPRM1*, *SLC2A9*, *MAPKAPK3*, *SCN1A*, *ITPKB*, *CHI3L1*, *PADI2*, and *MGST3*, several of which were present in all patients (frequency = 10). Among somatic mutations, *TERT* was the most frequently altered gene (detected in 50% of tumors), followed by *TP53*, *CDKN2A*, and *NF2*. Additional somatic alterations were observed at lower frequencies.

Recurrent germline variants were observed across multiple genes, with several variants present in all 10 individuals (frequency = 10). Frequently represented genes included: *XRCC1*, *CDKN2B-AS1*, *ABCB1*, *OPRM1*, *SLC2A9*, *MAPKAPK3*, *SCN1A*, *ITPKB*, *CHI3L1*, *PADI2* and *MGST3* (Figure 3).

**Figure 4:**
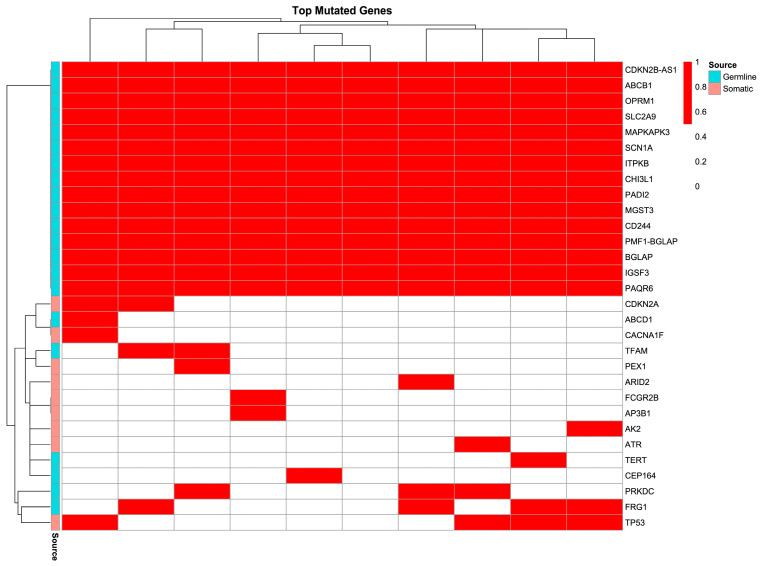
Heatmap of top mutated genes across germline and somatic compartments. Hierarchical clustering heatmap representing the presence or absence of top mutated genes across samples. Germline-dominant genes cluster separately from somatic-enriched genes, demonstrating distinct mutational patterns between constitutional and tumor-specific alterations. Canonical tumor suppressor genes such as *TP53* and *CDKN2A* form part of the somatic-enriched cluster, while recurrent germline variants show uniform distribution across individuals.

As shown in the heatmap of top mutated genes (Figure 4), many germline variants were consistently present across samples, suggesting shared population-level or ancestry-related variants rather than tumor-specific drivers.

### 3.4. Recurrent Somatic Mutations

Somatic mutation analysis identified recurrent alterations in canonical cancer-associated genes. The most frequently mutated somatic genes included: *TERT* (observed in 50% patients), followed by *TP53*, *CDKN2A* and *NF2* (Figure 3). Additional somatic variants were detected in *PRKDC*, *PEX1*, *TFAM*, *ABCD1*, *ARID2*, *ATR*, *AK2*, *FRG1*, and others, generally at lower frequencies (1–3 samples). Top mutated genes demonstrate distinct segregation between germline-dominant and somatic-dominant genes, with tumor suppressor genes such as *TP53* and *CDKN2A* forming part of the somatic-enriched cluster. Notably, *TERT* exhibited the highest recurrence among tumor samples, consistent with previously reported involvement of *TERT* alterations in telomere-related oncogenic pathways in OSCC. Notably, the detected *TERT* alterations corresponded to coding-region exonic variants identified within WES capture regions and do not represent canonical *TERT* promoter mutations.

### 3.5. Comparative Landscape of Germline and Somatic Alterations

Comparative analysis of germline and somatic alterations demonstrated a substantially higher background burden of recurrent germline-associated variants relative to the comparatively limited number of tumor-specific somatic mutations identified within the cohort. As expected, germline variants substantially outnumbered somatic coding alterations across all individuals, reflecting the broader background constitutional variation relative to tumor-specific acquired mutations. While most recurrent germline variants were also observed in adjacent normal tissues and likely represent recurrent population-associated variants, somatic alterations were primarily restricted to established cancer-associated genes involved in cell cycle regulation, telomere maintenance, and genomic stability. Furthermore, only limited overlap was observed between germline-associated and somatic mutation profiles. These observations suggest differing distributions of recurrent population-associated variants and tumor-specific coding alterations within the analyzed cohort; however, their biological relevance requires further validation in larger studies.

### 3.6. Comparative Mutational Landscape Analysis with 66 OSCC Patients gnomAD

To assess the recurrence and broader population relevance of the variants identified in the fresh biopsy cohort, a comparative analysis was performed using a previously characterized regional dataset comprising a total of 66 OSCC patients from the same geographical population [10,11]. This combined dataset included the 10 fresh biopsy whole-exome sequenced cases analyzed in the present study together with 56 additional previously analyzed OSCC samples, thereby enabling evaluation of regional recurrence patterns across a larger cohort (Supplementary Table S1).

Several variants demonstrated high recurrence across the 66-patient cohort. The most prevalent variant, rs708776 in the *ITPKB* gene, was detected in 98.48% of patients (65/66), followed by rs25487 in *XRCC1*, which occurred in 83.33% of patients (55/66). Additional recurrent variants included rs6667260 (*ITPKB*) observed in 80.30% of patients, rs1169288 (*HNF1A*) detected in 66.67%, and rs2075572 (*OPRM1*) present in 50% of the cohort. According to ClinVar annotations, several of these variants are classified as likely pathogenic or associated with drug response, indicating previously reported clinical or pharmacogenomic annotations that may warrant further investigation in OSCC. We note here that ClinVar pathogenicity annotations reflect previously reported clinical associations and should not be interpreted as definitive evidence of pathogenicity in OSCC.

Variants such as rs708776 and rs6667260 in *ITPKB*, rs25487 in *XRCC1*, rs1169288 in *HNF1A* and rs2075572 in *OPRM1* also display relatively high frequencies in South Asian population datasets, indicating that they may represent recurrent population-associated variants with possible pharmacogenomic relevance, rather than tumor-specific driver mutations (Supplementary Table S1).

Conversely, drug response SNV rs650245 in *OPRM1* (South Asian frequency: 1.593 × 10^−5^), pathogenic SNVs such as rs61730489 in *IGSF3* (1.159 × 10^−5^), rs76203768 in *CTBP2* (2.319 × 10^−5^), rs78386506 and rs77830704 in *CNN2* (2.352 × 10^−5^ and nearly absent, respectively), rs775321736 in *CDC27* (1.163 × 10^−5^), and rs758487568 in *POLE* (1.159 × 10^−5^), and pathogenic DELIS such as rs71709231 in *DBT* (0.002627), rs3839339 in *KLHL3* (0.006039), and rs753526329 in *AR* (0.0006575) were detected in >40% of the cohort despite being exceptionally rare in South Asian as well as global populations (Supplementary Table S1). Their enrichment within the study group suggests possible regional enrichment patterns that require validation in larger independent cohorts.

**Figure 5:**
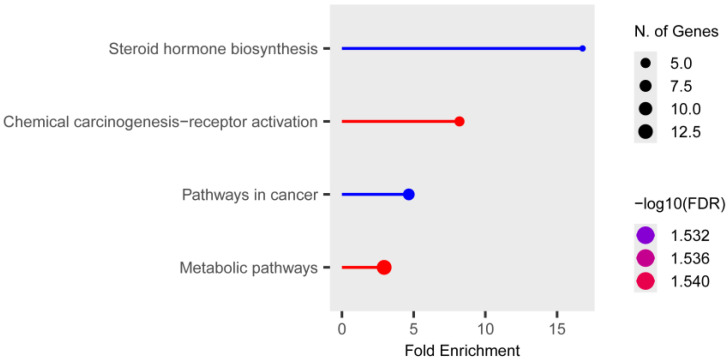
KEGG pathway enrichment analysis of recurrent genes identified in the 66-patient OSCC cohort. KEGG Pathway Analysis was performed using ShinyGO v0.85.1 web-based server (https://bioinformatics.sdstate.edu/go/, Last accessed on 4 March 2026).

Genes harboring recurrent variants in the 66-patient dataset, including *ITPKB*, *XRCC1*, *HNF1A*, and *OPRM1*, are functionally linked to pathways involved in DNA repair, immune signaling, metabolic regulation, and cellular stress responses, all of which have been implicated in oral carcinogenesis (Figure 5). Notably, the *XRCC1* variant rs25487, widely reported as a drug response–associated polymorphism, has been implicated in modulating cellular responses to DNA-damaging chemotherapeutic agents and radiotherapy, owing to its role in the base excision repair (BER) pathway. Variants in *OPRM1*, such as rs2075572, are also linked to drug response phenotypes, particularly influencing opioid signaling and pain management, which may have implications for supportive care in cancer patients. The presence of such pharmacogenomic variants highlights the potential influence of host genetic background on treatment response and clinical management of OSCC.

KEGG pathway enrichment analysis of recurrent genes identified enrichment of pathways related to cancer, chemical carcinogenesis, steroid hormone biosynthesis, metabolic regulation, and xenobiotic response, supporting the potential involvement of candidate population-associated variants and environmental interaction pathways in OSCC.

Tumor–normal comparison within the dataset revealed that many recurrent variants were present in both tumor and adjacent normal tissues, indicating that they likely represent germline polymorphisms rather than tumor-acquired somatic mutations. This observation aligns with findings from the fresh biopsy sequencing analysis, where a substantial proportion of variants were similarly detected in matched normal tissues. These results suggest that several recurrent variants may represent population-associated genetic markers that warrant further investigation in relation to disease heterogeneity and treatment variability. Furthermore, comparison with The Cancer Genome Atlas (TCGA) datasets indicated limited representation of several of these variants, supporting the possibility that the mutational profile observed in this southern Indian OSCC population reflects region-specific genetic architecture not fully captured in global cancer genomic studies.

Overall, integration of the fresh biopsy sequencing results with the larger 66-patient dataset reveals a consistent pattern of highly recurrent variants across cohorts. These findings reinforce the presence of a core set of population-associated genetic variants within OSCC patients from the southwest coast of India, highlighting recurrent population-associated variants observed within this regional cohort that may warrant further investigation in relation to disease heterogeneity and therapeutic response.

## 4. Discussion

The present study provides a comprehensive genomic characterization of oral squamous cell carcinoma (OSCC) using whole-exome sequencing (WES) of fresh biopsy specimens. By integrating fresh tissue sequencing with previously reported FFPE-based genomic data from the same population, this study highlights the presence of a conserved set of genetic variants that may represent a stable genomic framework of OSCC in this region. Importantly, the use of fresh biopsy tissues enabled higher-fidelity variant detection while simultaneously validating genomic signals previously observed in archival FFPE samples, thereby strengthening the biological robustness of the findings.

One of the most notable observations in this study is the markedly higher burden of germline variants compared with somatic mutations. Although classical tumor-specific driver mutations such as *TP53*, *CDKN2A*, and *TERT* were identified, the overall somatic mutation burden remained relatively low. Similar patterns of relatively low somatic mutational burden have been reported in subsets of OSCC across different exposure backgrounds. Large-scale genomic studies such as The Cancer Genome Atlas (TCGA) have demonstrated recurrent somatic alterations in *TP53*, *FAT1*, *CDKN2A*, *NOTCH1*, and *PIK3CA*, although substantial variability exists across populations and exposure profiles [10,11,26,27]. The restricted somatic mutation spectrum observed in this cohort may reflect the comparatively limited number of detectable coding alterations identified within the analyzed samples; however, larger studies will be required to determine whether this pattern is reproducible in regional OSCC populations.

Importantly, several highly recurrent variants identified in genes such as *XRCC1*, *ITPKB*, and *OPRM1* demonstrated high frequencies within South Asian population datasets, suggesting that these variants may represent recurrent population-associated or pharmacogenomic variants rather than tumor-specific somatic alterations. In contrast, a subset of recurrent variants including rs650245 (*OPRM1*), rs61730489 (*IGSF3*), rs76203768 (*CTBP2*), rs78386506/rs77830704 (*CNN2*), rs775321736 (*CDC27*), rs758487568 (*POLE*), rs71709231 (*DBT*), rs3839339 (*KLHL3*), and rs753526329 (*AR*) exhibited extremely low South Asian and global population frequencies despite recurring within the cohort, suggesting potential recurrence patterns that warrant investigation in larger regional cohorts, requiring validation in larger studies.

*XRCC1*, *ITPKB*, *ABCB1*, *OPRM1*, *CHI3L1*, and *MAPKAPK3* converge on biologically relevant pathways involving DNA repair, immune signaling, inflammatory responses, and xenobiotic metabolism. *XRCC1*, a critical component of the base excision repair pathway, plays an essential role in repairing oxidative DNA damage. Polymorphisms in *XRCC1* have been associated with increased susceptibility to head and neck cancers and altered responses to DNA-damaging therapies [28,29], supporting the possibility that DNA repair–related variation may influence inter-individual differences in response to carcinogenic exposure.

Similarly, *ABCB1* encodes the ATP-binding cassette transporter P-glycoprotein, which regulates xenobiotic efflux and drug transport. Genetic variation in *ABCB1* has been linked to altered pharmacokinetics and multidrug resistance in several cancers [30]. The recurrent detection of *ABCB1* variants may warrant further investigation regarding potential roles in xenobiotic metabolism and pharmacogenomic variability.

In addition to DNA repair and drug transport pathways, several recurrent variants identified in this study are associated with immune and inflammatory signaling. ITPKB is involved in calcium-mediated signaling pathways regulating immune activation, while CHI3L1 (YKL-40) has been implicated in chronic inflammation, extracellular matrix remodeling, and tumor microenvironment regulation [31]. MAPKAPK3, a downstream effector of the MAP kinase pathway, participates in cellular stress and inflammatory responses [32]. Collectively, these findings highlight recurrent variation in immune–inflammatory pathway–related genes within this cohort.

Taken together, these observations highlight recurrent variation in pathways related to DNA repair (*XRCC1*), xenobiotic metabolism (*ABCB1*), and immune signaling (*ITPKB*, *CHI3L1*, and *MAPKAPK3*) within this cohort. Further functional and population-scale studies will be necessary to determine whether these pathways contribute to OSCC susceptibility or progression in this regional population.

An important observation in this study is the high recurrence of several germline variants when compared with a previously analyzed regional cohort. Although adjacent normal tissues were used as controls, the possibility of molecular alterations arising from field cancerization cannot be completely excluded. However, paired tumor–normal comparative analysis and annotation against population databases enabled comparative classification of recurrent tumor-associated and shared variants within the limitations of adjacent tissue controls. The reproducibility of variants such as *rs708776* (*ITPKB*) and *rs25487* (*XRCC1*) across independent cohorts supports the recurrence of several population-associated variants across independent regional cohorts. Notably, several of these variants are underrepresented in global datasets such as TCGA, emphasizing the importance of region-specific genomic investigations [26].

The identification of recurrent somatic mutations in *TERT*, *TP53*, and *CDKN2A* further underscores the role of telomere maintenance and cell cycle dysregulation in OSCC. Activating alterations in *TERT* promote telomerase activation and replicative immortality [33], while *TP53* mutations contribute to genomic instability and tumor progression [33]. These findings suggest that recurrent population-associated variants and canonical somatic alterations coexist within the analyzed cohort.

The use of fresh biopsy tissues represents a key strength of this study. Formalin fixation can introduce sequencing artefacts and DNA fragmentation, whereas fresh tissue preserves nucleic acid integrity and improves detection accuracy [34]. The concordance between fresh biopsy data and previously reported FFPE datasets further validates the robustness of the observed genomic patterns.

From a translational perspective, the identification of recurrent pharmacogenomic variants suggests that host genetic background may influence therapeutic response. Variants in genes such as *ABCB1* and *XRCC1* have been associated with altered sensitivity to chemotherapy and radiotherapy [35]. These observations may provide a rationale for future investigation of pharmacogenomic markers in larger clinically annotated cohorts and can support personalized therapeutic strategies [35,36].

Despite these insights, several limitations should be acknowledged. We note here that the present study was designed primarily as an exploratory genomic profiling effort in an underrepresented high-risk regional population rather than as a definitive biomarker discovery study. The relatively small whole-exome sequencing cohort limits statistical generalizability and may reduce sensitivity for detecting low-frequency somatic alterations. However, integration with an independent regional cohort comprising 66 OSCC patients enabled assessment of recurrence patterns and provided additional support for the reproducibility of several recurrent variants identified in this population. Tumor purity estimation and orthogonal validation of variant allele frequencies were not performed and therefore low-frequency somatic variants may have been under-detected. In addition, whole-exome sequencing primarily targets coding regions and does not capture regulatory variants in non-coding regions. Functional validation studies were beyond the scope of this work and are required to establish the biological significance of identified variants. Future studies incorporating larger multi-center cohorts and integrative multi-omics approaches will be essential to further elucidate the molecular mechanisms underlying OSCC. We note here that, given the exploratory nature of the study and the limited number of tumor samples analyzed, the findings should not be interpreted as definitive evidence of OSCC-specific driver events or clinical biomarkers. Accordingly, all biological and clinical interpretations presented in this study should be considered preliminary.

## 5. Conclusions

In conclusion, this study provides a preliminary population-specific genomic characterization of OSCC from the southwest coast of India using whole-exome sequencing of paired tumor and adjacent normal tissues. The findings indicate the presence of recurrent germline-associated variants alongside a limited spectrum of canonical somatic alterations involving genes such as *TP53*, *CDKN2A*, and *TERT*. Several recurrent variants were also observed across an independent regional cohort and were enriched in pathways related to DNA repair, immune signaling, carcinogen response, and pharmacogenomic regulation. Notably, a subset of recurrent germline-associated variants, in *OPRM1*, *IGSF3*, *CTBP2*, *CNN2*, and *CDC27* demonstrated extremely low South Asian and global population frequencies despite recurring within the cohort, suggesting possible recurrence patterns that warrant evaluation in larger regional studies that warrants further investigation. Given the limited sample size, use of adjacent normal tissues as controls, and absence of functional validation, these findings should be considered exploratory and hypothesis-generating. Larger multi-center studies and functional analyses will be necessary to determine the biological and clinical relevance of these variants in OSCC.

## Figures and Tables

**Table 1:** **Clinical and demographic characteristics of the fresh biopsy OSCC cohort.** Summary of demographic and clinical parameters of the ten patients recruited for whole exome sequencing, including age, sex, tumor site, clinical stage, and relevant exposure history.

Sl. No	Hospital No	Site	Age/Sex	Geographical Location	Language	Family History	Habits	Primary Treatment Modality	Stage	Sample ID (Neuberg)
**1**	5112049/K73803	Lateral border of tongue	39/M	Haveri	Kannada	NRFH	Tobacco, alcohol	Surgery	T3N1Mx	1C-FBYEN, 11N-FBYEN
**2**	5112566/K74347	Right gingivobuccal sulcus	38/M	Kedila, Mangalore	Kannada, Malayalam	HCC (father’s sister)	Gutkha, smoking	Surgery	T4aN1Mx	2C-FBYEN, 12N-FBYEN
**3**	4809819/K81321	Right buccal mucosa	68/F	Uttar Kannada	Kannada	NRFH	Pan chewing	Surgery	T3N0Mx	4C-FBYEN, 14N-FBYEN
**4**	5173170/K87722	Right border of tongue	61/M	Kannur	Malayalam	NRFH	Tobacco (smoking, chewing)	Surgery	T2N1Mx	5C-FBYEN, 15N-FBYEN
**5**	5171431/K87442	Lower alveolus	60/M	Puttur	Kannada	NRFH	Tobacco chewing	Surgery	T4N1Mx	6C-FBYEN, 16N-FBYEN
**6**	5189742/K90985	Right buccal mucosa	63/F	Puttur	Kannada	NRFH	Tobacco chewing, smoking	Surgery	T1N0Mx	7C-FBYEN, 17N-FBYEN
**7**	5187998	Left retromolar trigone	71/F	Kasaragod	Malayalam, English	NRFH	Tobacco chewing	Surgery	T3N2Mx	8C-FBYEN, 18N-FBYEN
**8**	5185106/K92029	Right gingivobuccal sulcus	57/M	Shimoga	Kannada	Mother–uterine cancer	Tobacco chewing	Surgery	T4aN1Mx	9C-FBYEN, 19N-FBYEN
**9**	5193475/K93797	Right upper alveolus	46/F	Belthangady	Kannada, Byari	NRFH	Pan chewing, snuff	Surgery	T2N0Mx	10C-FBYEN, 20N-FBYEN
**10**	5189070/K96722	Lower alveolus	62/F	Chitradurga	Kannada	NRFH	Tobacco chewing	Surgery (recurrence)	T2N1Mx	21C-FBYEN, 22N-FBYEN

This table presents the clinical and demographic profile of patients included in the fresh biopsy cohort analyzed by whole-exome sequencing. Variables include tumor site, age, sex, geographical origin, language, family history, risk habits, primary treatment modality, and TNM-based clinical staging. Paired tumor and adjacent normal tissues were collected for each case wherever available to enable comparative genomic analysis. Abbreviations: NRFH, no relevant family history; HCC, hepatocellular carcinoma; TNM, tumor–node–metastasis classification; C, tumor sample; N, adjacent normal tissue.

## Data Availability

The original contributions presented in this study are included in the article/Supplementary Material. Further inquiries can be directed to the corresponding authors.

## Supplementary Materials

The following supporting information can be downloaded at https://www.mdpi.com/article/10.3390/biomedicines14061346/s1, Table S1: Comparative Mutational Landscape Analysis with 66 OSCC patients.
