# Supplementary material for: Whole-Exome Sequencing Identifies Recurrent Germline-Associated and Somatic Variants in Oral Squamous Cell Carcinoma from Southwest India
Source: Biomedicines. 2026 Jun 15;14(6):1346. doi: 10.3390/biomedicines14061346 (PMC13297059; doi:10.3390/biomedicines14061346)
Supplement: Supplementary file 1 [file biomedicines-14-01346-s001.zip › biomedicines-4278565-supplementary.pdf]

Supplementary Table S1: Comparative Mutational Landscape Analysis with 66 OSCC patients

**Variant**	**Gene**	**Mutation Type (dbSNP)**	**ClinVar Classification**	**Patient Count (N=66)**	**Frequency Percentage**	**TCGA**	**Overall Frequency (gnomAD)**	**South Asian Frequency (gnomAD)**	**DbGENVOK database**	**Normal vs Tumor comparison**
rs708776	*ITPKB*	SNV (Missense)	LIKELY PATHOGENIC	65	98.48	no	0.95	0.99	Present	Present in both Tumor and Normal Tissue
rs25487	*XRCC1*	SNV (coding sequence variant)	DRUG RESPONSE	55	83.33	no	0.65	0.65	Present	Present in both Tumor and Normal Tissue
rs6667260	*ITPKB*	SNV (Missense)	LIKELY PATHOGENIC	53	80.3	no	0.48	0.54	Present	Present in both Tumor and Normal Tissue
rs1169288	*HNF1A*	SNV (coding sequence variant)	Likely pathogenic(1)|Benign(10)	44	66.67	no	0.33	0.42	Present	Tumor and Normal comparison not done
rs2075572	*OPRM1*	SNV (genic downstream transcript variant)	drug response	33	50	no	0.59	0.73	Present	Present in both Tumor and Normal Tissue
rs562859	*OPRM1*	SNV (Missense)	drug response	33	50	no	0.68	0.82	Present	Present in both Tumor and Normal Tissue
rs650245	*OPRM1*	SNV (genic downstream transcript variant)	drug response	33	50	no	1.766e-06	1.593e-05	Present	Present in both Tumor and Normal Tissue
rs540825	*OPRM1*	SNV (Missense)	drug response	33	50	no	0.7679	0.8462	Present	Present in both Tumor and Normal Tissue
rs675026	*OPRM1*	SNV (genic downstream transcript variant)	drug response	33	50	no	0.6796	0.8224	Present	Present in both Tumor and

										Normal Tissue
rs9282821	*OPRM1*	SNV (intron variant)	drug response	33	50	no	0.5896	0.7308	Present	Present in both Tumor and Normal Tissue
rs9333378	*MGST3*	SNV (splice acceptor variant)	Association	33	50	no	0.6262	0.7705	Present	Present in both Tumor and Normal Tissue
rs61730489	*IGSF3*	SNV (stop gained)	Pathogenic	33	50	no	4.789e-06	1.159e-05	Present	Present in both Tumor and Normal Tissue
rs76203768	*CTBP2*	SNV (Missense)	Pathogenic	33	50	no	1.984e-05	2.319e-05	Present	Present in both Tumor and Normal Tissue
rs78386506	*CNN2*	SNV (Missense)	Pathogenic	33	50	no	1.381e-06	2.352e-05	Present	Present in both Tumor and Normal Tissue
rs77830704	*CNN2*	SNV (Missense)	Pathogenic	33	50	no	1.105e-05	0	Present	Present in both Tumor and Normal Tissue
rs775321736	*CDC27*	SNV (coding sequence variant)	Pathogenic	33	50	no	3.425e-06	1.163e-05	Present	Present in both Tumor and Normal Tissue
rs2214102	*ABCB1*	SNV (synonymous)	drug response	33	50	no	0.9264	0.9884	Present	Present in both Tumor and Normal Tissue
rs1211152	*ABCB1*	SNV (intron variant)	drug response	33	50	no	0.9393	0.9921	Present	Present in both Tumor and Normal Tissue
rs165728	*COMT*	SNV (downstream	drug response	33	50	no	0.9138	0.8805	Absent	Present in both Tumor and

		transcript variant)								Normal Tissue
rs509504	*PCSK9*	SNV (non coding transcript variant)	Conflicting interpretations of pathogenicity	33	50	no	0.9983	0.9998	Present	Tumor and Normal comparis on not done
rs4508371	*MYO1E*	SNV (intron variant)	Conflicting interpretations of pathogenicity	33	50	no	0.9981	0.9999	Present	Tumor and Normal comparis on not done
rs58528565	*KMT2C*	SNV (coding sequence variant)	Conflicting interpretations of pathogenicity	33	50	no	0	0	Present	Tumor and Normal comparis on not done
rs3842753	*INS-IGF2*	SNV (intron variant)	Conflicting interpretations of pathogenicity	33	50	no	0.7205	0.8271	Present	Tumor and Normal comparis on not done
rs3837692	*CLN6*	DELINS (3 prime UTR variant)	Conflicting interpretations of pathogenicity	33	50	no	0.4749	0.3375	Present	Tumor and Normal comparis on not done
rs55656324	*RELN*	DELINS (inframe insertion)	Conflicting interpretations of pathogenicity	33	50	no	0.02446	0.04283	Absent	Tumor and Normal comparis on not done
rs12460842	*PPAN-P2RY11*	SNV (2KB upstream variant)	Association	32	48.48	no	0.5827	0.6814	Present	Present in both Tumor and Normal Tissue
rs1551570	*PPAN*	SNV (2KB upstream variant)	Association	32	48.48	no	0.5869	0.6787	Absent	Present in both Tumor and Normal Tissue
rs3826784	*EIF3G*	SNV (intron variant)	Association	32	48.48	no	0.6092	0.6819	Present	Present in both Tumor and Normal Tissue
rs414171	*CISH*	SNV (2KB upstream variant)	risk factor	32	48.48	no	0.843	0.908	Absent	Present in both Tumor and

										Normal Tissue
rs4950928	*CHI3L1*	SNV (upstream transcript variant)	risk factor	32	48.48	no	0.7949	0.8079	Absent	Present in both Tumor and Normal Tissue
rs1801214	*WFS1*	SNV (synonymous)	Conflicting interpretations of pathogenicity	32	48.48	no	0.6232	0.6951	Present	Tumor and Normal comparison not done
rs758487568	*POLE*	DELINS (coding sequence variant)	Pathogenic(1)|Uncertain significance(1)	32	48.48	no	3.763e-05	1.159e-05	Present	Tumor and Normal comparison not done
rs5885806	*PEX1*	DELINS (intron variant)	Conflicting interpretations of pathogenicity	32	48.48	no	0.07706	0.1028	Present	Tumor and Normal comparison not done
rs111493987	*KMT2C*	DELINS (intron variant)	Conflicting interpretations of pathogenicity	32	48.48	no	0	0	Present	Tumor and Normal comparison not done
rs3180234	*IL36RN*	SNV (3 prime UTR variant)	Conflicting interpretations of pathogenicity	32	48.48	no	0.6596	0.8001	Present	Tumor and Normal comparison not done
rs3180235	*IL36RN*	SNV (3 prime UTR variant)	Conflicting interpretations of pathogenicity	32	48.48	no	0.6666	0.8049	Present	Tumor and Normal comparison not done
rs3839918	*GATA3*	DELINS (3 prime UTR variant)	Conflicting interpretations of pathogenicity	32	48.48	no	0.5994	0.6001	Present	Tumor and Normal comparison not done
rs70983380	*GARS1*	DELINS (3 prime UTR variant)	Conflicting interpretations of pathogenicity	32	48.48	no	0.4481	0.3583	Absent	Tumor and Normal comparison not done
rs71709231	*DBT*	DELINS (3 prime UTR variant)	Conflicting interpretations of pathogenicity	32	48.48	no	0.007293	0.002627	Present	Tumor and Normal comparis

										on not done
rs11307362	*ACADSB*	DELINS (intron variant)	Conflicting interpretations of pathogenicity	32	48.48	no	0.7759	0.8403	Absent	Tumor and Normal comparis on not done
rs759330	*PAQR6*	DELINS (non coding transcript variant)	Association	31	46.97	no	0.7319	0.7774	Absent	Present in both Tumor and Normal Tissue
rs71021021	*OPRM1*	DELINS (3 prime UTR variant)	drug response	31	46.97	no	0.8689	0.9307	Present	Present in both Tumor and Normal Tissue
rs727479	*MIR4713 HG*	SNV (intron variant)	drug response	31	46.97	no	0.6677	0.7376	Absent	Present in both Tumor and Normal Tissue
rs7417106	*PERM1*	SNV (Missense)	Pathogenic	31	46.97	no	0.792	0.7746	Absent	Present in both Tumor and Normal Tissue
rs1801206	*WFS1*	SNV (synonymo us)	Conflicting interpretations of pathogenicity	31	46.97	no	0.6051	0.6353	Present	Tumor and Normal comparis on not done
rs71329437	*VLDLR-AS1*	DELINS (5 prime UTR variant)	Conflicting interpretations of pathogenicity	31	46.97	no	0.3801	0.2349	Absent	Tumor and Normal comparis on not done
rs2228570	*VDR*	SNV (coding sequence variant)	Conflicting interpretations of pathogenicity	31	46.97	no	0.6253	0.766	Present	Tumor and Normal comparis on not done
rs35597368	*PDGFRA*	SNV (coding sequence variant)	Likely pathogenic(1)|Benig n(6)	31	46.97	no	0.1218	0.2048	Present	Tumor and Normal comparis on not done
rs712701	*PAX4*	SNV (Missense)	Conflicting interpretations of pathogenicity	31	46.97	no	0.7674	0.7429	Present	Tumor and Normal comparis

										on not done
rs11300320	*GALC*	DELINS (intron variant)	Conflicting interpretations of pathogenicity	31	46.97	no	0.9669	0.9725	Present	Tumor and Normal comparis on not done
rs3127334	*TBXT*	SNV (intron variant)	risk factor	30	45.45	no	0.742	0.746	Absent	Present in both Tumor and Normal Tissue
rs2305795	*PPAN-P2RY11*	SNV (500B downstrea m variant)	Association	30	45.45	no	0.573	0.6325	Present	Present in both Tumor and Normal Tissue
rs1801253	*ADRB1*	SNV (coding sequence variant)	association|drug response	30	45.45	no	0.7344	0.7554	Present	Present in both Tumor and Normal Tissue
rs67705775	*MTR*	DELINS (3 prime UTR variant)	Conflicting interpretations of pathogenicity	30	45.45	no	0.3806	0.414	Present	Tumor and Normal comparis on not done
rs201268947	*GFPT1*	DELINS (intron variant)	Conflicting interpretations of pathogenicity	30	45.45	no	0.4721	0.4329	Present	Tumor and Normal comparis on not done
rs56062620	*ESCO2*	DELINS (intron variant)	Conflicting interpretations of pathogenicity	30	45.45	no	0.4041	0.3697	Present	Tumor and Normal comparis on not done
rs4727853	*CFTR-AS1*	SNV (intron variant)	Conflicting interpretations of pathogenicity	30	45.45	no	0.002552	0.002021	Absent	Tumor and Normal comparis on not done
rs7294	*VKORC1*	SNV (3 prime UTR variant)	drug response	29	43.94	no	0.3914	0.6877	Present	Present in both Tumor and Normal Tissue
rs497332	*OPRM1*	SNV (genic downstrea m	drug response	29	43.94	no	0.9177	0.9756	Present	Present in both Tumor and

		transcript variant)								Normal Tissue
rs1202168	*ABCB1*	SNV (intron variant)	drug response	29	43.94	no	0.4549	0.6096	Present	Present in both Tumor and Normal Tissue
rs10789501	*CYP4A22*	SNV (intron variant)	Association	29	43.94	no	0.3924	0.6268	Absent	Present in both Tumor and Normal Tissue
rs2245425	*TOR1AIP 1*	SNV (splice acceptor variant)	Conflicting interpretations of pathogenicity	29	43.94	no	0.6269	0.6858	Present	Tumor and Normal comparis on not done
rs4880	*SOD2*	SNV (coding sequence variant)	Conflicting interpretations of pathogenicity|risk factor	29	43.94	no	0.4921	0.5191	Present	Tumor and Normal comparis on not done
rs4149095	*SLCO1B1*	DELINS (intron variant)	Conflicting interpretations of pathogenicity	29	43.94	no	0.3998	0.4684	Present	Tumor and Normal comparis on not done
rs698761	*PREPL*	SNV (Missense)	Conflicting interpretations of pathogenicity	29	43.94	no	0.6599	0.6645	Present	Tumor and Normal comparis on not done
rs3839339	*KLHL3*	DELINS (3 prime UTR variant)	Conflicting interpretations of pathogenicity	29	43.94	no	0.001219	0.006039	Present	Tumor and Normal comparis on not done
rs39802851 2	*IGF1R*	DELINS (3 prime UTR variant)	Conflicting interpretations of pathogenicity	29	43.94	no	0.5886	0.6458	Present	Tumor and Normal comparis on not done
rs75856790 6	*COL3A1*	DELINS (intron variant)	Conflicting interpretations of pathogenicity	29	43.94	no	0.194	0.09859	Present	Tumor and Normal comparis on not done
rs1061170	*CFH*	SNV (non coding transcript variant)	Conflicting interpretations of pathogenicity	29	43.94	no	0.6361	0.6942	Present	Tumor and Normal comparis

										on not done
rs11277519	*ADAMTS 17*	DELINS (3 prime UTR variant)	Conflicting interpretations of pathogenicity	29	43.94	no	0.6338	0.5808	Present	Tumor and Normal comparis on not done
rs7439366	*UGT2B7*	SNV (coding sequence variant)	drug response	28	42.42	no	0.4938	0.567	Present	Present in both Tumor and Normal Tissue
rs20541	*IL13*	SNV (coding sequence variant)	risk factor	28	42.42	no	0.7864	0.7092	Present	Present in both Tumor and Normal Tissue
rs1063192	*CDKN2B*	SNV (3 prime UTR variant)	Protective	28	42.42	no	0.6518	0.7125	Present	Present in both Tumor and Normal Tissue
rs57078153	*TBX3*	DELINS (5 prime UTR variant)	Conflicting interpretations of pathogenicity	28	42.42	no	0.3109	0.2265	Present	Tumor and Normal comparis on not done
rs771282908	*GABRG2*	DELINS (intron variant)	Conflicting interpretations of pathogenicity	28	42.42	no	0.1155	0.1202	Present	Tumor and Normal comparis on not done
rs6467	*CYP21A2*	SNV (5 prime UTR variant)	Conflicting interpretations of pathogenicity	28	42.42	no	0.595	0.6165	Present	Tumor and Normal comparis on not done
rs199511358	*CEP290*	DELINS (genic upstream transcript variant)	Conflicting interpretations of pathogenicity	28	42.42	no	0.1351	0.1545	Present	Tumor and Normal comparis on not done
rs753526329	*AR*	DELINS (coding sequence variant)	PATHOGENIC	28	42.42	no	7.435e-05	0.0006575	Present	Tumor and Normal comparis on not done
rs2294918	*PNPLA3*	SNV (coding sequence variant)	Conflicting interpretations of pathogenicity	28	42.42	no	0.6218	0.7308	Absent	Tumor and Normal comparis

										on not done
rs2269577	*XBP1*	SNV (upstream transcript variant)	risk factor	27	40.91	no	0.3644	0.5053	Present	Present in both Tumor and Normal Tissue
rs7668258	*UGT2B7*	SNV (upstream transcript variant)	drug response	27	40.91	no	0.494	0.5692	Present	Present in both Tumor and Normal Tissue
rs7116432	*CD44*	SNV (3 prime UTR variant)	Association	27	40.91	no	0.3513	0.5326	Present	Present in both Tumor and Normal Tissue
rs2070635	*AHSG*	SNV (intron variant)	Association	27	40.91	no	0.4459	0.5253	Present	Present in both Tumor and Normal Tissue
rs60544332	*MYH11*	DELINS (intron variant)	Conflicting interpretations of pathogenicity	27	40.91	no	0.1587	0.1395	Present	Tumor and Normal comparis on not done
rs10117	*HSPA9*	SNV (coding sequence variant)	Conflicting interpretations of pathogenicity	27	40.91	no	0.4321	0.6371	Present	Tumor and Normal comparis on not done
rs6030	*F5*	SNV (Missense)	Conflicting interpretations of pathogenicity	27	40.91	no	0.332	0.4015	Present	Tumor and Normal comparis on not done
rs71379679	*COG7*	DELINS (intron variant)	Conflicting interpretations of pathogenicity	27	40.91	no	0.354	0.3803	Present	Tumor and Normal comparis on not done
rs1553745484	-	DELINS (inframe insertion)	Conflicting interpretations of pathogenicity	27	40.91	no	0.3806	0.5356	Absent	Tumor and Normal comparis on not done

Germline variants with population frequency <1% are highlighted in red. The variants with frequency ~1% are highlighted in blue
